# Past, present and future distribution of the yellow fever mosquito *Aedes aegypti*: The European paradox

**DOI:** 10.1016/j.scitotenv.2022.157566

**Published:** 2022-11-15

**Authors:** William Wint, Peter Jones, Moritz Kraemer, Neil Alexander, Francis Schaffner

**Affiliations:** a ERGO – Environmental Research Group Oxford, c/o Department Zoology, Mansfield Road, Oxford OX1 3SZ, United Kingdom; b Waen Associates, Y Waen, Islaw'r Dref, Dolgellau, Gwynedd LL40 1TS, United Kingdom; c University of Oxford, Department of Zoology, Peter Medawar Building For Pathogen Research, 3 S Parks Rd, Oxford OX1 3SY, United Kingdom; d Francis Schaffner Consultancy, Lörracherstrasse 50, 4215 Riehen, Switzerland; e National Centre for Vector Entomology, Institute of Parasitology, Vetsuisse Faculty, University of Zürich, Winterthurerstrasse 266a, 8057 Zürich, Switzerland

**Keywords:** Vector-borne diseases, Historical distribution, Spatial modelling, Western Palaearctic region, Culicidae

## Abstract

The global distribution of the yellow fever mosquito *Aedes aegypti* is the subject of considerable attention because of its pivotal role as a biological vector of several high profile disease pathogens including dengue, chikungunya, yellow fever, and Zika viruses. There is also a lot of interest in the projected future species' distribution. However, less effort has been focused on its historical distribution, which has changed substantially over the past 100 years, especially in southern Europe where it was once widespread, but largely disappeared by the middle of the 20th century.

The present work utilises all available historical records of the distribution of *Ae. aegypti* in southern Europe, the Near East within the Mediterranean Basin and North Africa from the late 19th century until the 1960's to construct a spatial distribution model using matching historical climatic and demographic data.

The resulting model was then implemented using current climate and demographic data to assess the potential distribution of the vector in the present. The models were rerun with several different assumptions about the thresholds that determine habitat suitability for *Ae. aegypti*. The historical model matches the historical distributions well. When it is run with current climate values, the predicted present day distribution is somewhat broader than it used to be particularly in north-west France, North Africa and Turkey. Though it is beginning to reappear in the eastern Caucasus, this ‘potential’ distribution clearly does not match the actual distribution of the species, which suggests some other factors are responsible for its absence. Future distributions based on the historical model also do not match future distributions derived from models based only on present day vector distributions, which predict little or no presence in the Mediterranean Region. At the same time, the vector is widespread in the USA which is predicted to consolidate its range there in future. This contradiction and the implication for possible re-invasion of Europe are discussed.

## Highlights

• *Aedes aegypti* was historically widespread on the European continent but is now largely absent.
• Spatial models of historical distribution, with matching contemporaneous data fit distribution well.
• Historical model projected to 2015 matches USA distribution, & predicts wide presence in Europe.
• Reasons for absence in Europe are discussed, but remain uncertain.

## Graphical abstract

**Figure:**
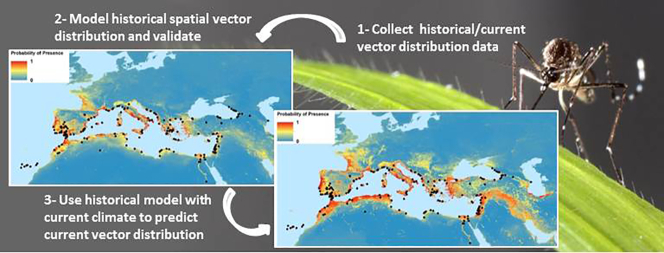

## 1 Introduction

The global distribution of the yellow fever mosquito *Aedes* (*Stegomyia*) *aegypti* (Linnaeus, 1762) is the subject of considerable attention because of its pivotal role as a biological vector of several high profile disease pathogens including dengue, chikungunya, yellow fever, and Zika viruses (Kraemer et al., 2015a; Schaffner and Mathis, 2014). Among the two recognised taxonomic forms, *Ae. aegypti aegypti* and *Ae. aegypti formosus*, the former (herein referred to as *Ae. aegypti*) is highly invasive and has become widespread, with potential to expand its distribution in the future (European Centre for Disease Prevention and Control, 2012; Kearney et al., 2009; Kraemer et al., 2015b). There is evidence that it originated from Africa where it became “domesticated”, i.e., switched to use human-generated water containers for larval development and humans as blood source (Powell et al., 2018). Its intercontinental spread around the tropical and subtropical world started with the rise of transatlantic shipping in the sixteenth century, which was followed by worldwide epidemics of diseases caused by pathogens transmitted by *Ae. aegypti* (Powell and Tabachnick, 2013). At the apogee of its distribution in Europe, during the early 20th century, *Ae. aegypti* had well established populations in the whole Mediterranean Basin (Christophers, 1960; Schaffner and Mathis, 2014). It was sometimes reported to be highly abundant (France: (Blanchard, 1917); Greece: (Blanc and Caminopetros, 1930); Italy: (La Face and Raffaele, 1928); Russia: (Marzinowsky, 1914); Portugal: (Sarmento and França, 1902)) and present over long periods in coastal and inland areas, far from Points of Entry (Spain: (Gil Collado, 1930); Greece: (Blanc and Caminopetros, 1930)). Since the other species suspected to contribute to dengue transmission *Ae. (Fredwardsius) vittatus* (Bigot, 1861) and *Ae. (Stegomyia) cretinus* Edwards, 1921 had only sporadic and patchy distributions, *Ae. aegypti* can reliably be incriminated as the responsible vector for significant epidemics of yellow fever in e.g. Spain, 1819-24, and dengue in e.g. Greece, 1927-28 (Hoffmann, 1931).

The mosquito almost disappeared from its western Palaearctic range by the 1960's, as a result of dedicated control campaigns (Marzinowsky, 1930) or possibly as a side effect of malaria vector control (Holstein, 1967). It is also suspected that the introduction of piped water to rural villages, and the consequent reduction in potential breeding sites (Blanc and Caminopetros, 1930; Holstein, 1967) contributed to the vector disappearance. No significant established population was reported between 1960 and 2000 but a few sporadic presence records exist (Schaffner and Mathis, 2014), and occurrence of remnant populations at some locations is suggested (Kotsakiozi et al., 2018). *Aedes aegypti* is now sporadically reported at Points of Entry (as defined by WHO International Health Regulation) like airports (Ibáñez-Justicia et al., 2020), or sea ports (Jeannin et al., 2019), but also imported used tyre depots (Scholte et al., 2010), or private hothouses (Kampen et al., 2016). Establishment of *Ae. aegypti* has recently been reported from Madeira (Almeida et al., 2007), southern Egypt (Abozeid et al., 2018) and the Caucasus (Yunicheva et al., 2008), and it has been spreading west along the Black Sea coast in Turkey (Akiner et al., 2016) and Crimea (Ganushkina et al., 2020) but has not yet established anywhere in the Mediterranean Basin.

Globally, particular features that have been associated with the presence of *Ae. aegypti* include urbanisation, socioeconomic factors, building design and construction features, the quality of water supply and management, and the quality of other public health infrastructure services (Jansen and Beebe, 2010). Overall, the geographical distribution of *Ae. aegypti* is not static, and appears to have undergone significant changes over time on several continents. In the Americas, large and coordinated mosquito eradication efforts were implemented following the 1947 resolution by member nations of the Pan American Health Organisation (PAHO). These resulted in a marked decline in *Ae. aegypti* populations in that part of the world and the subsequent successful eradication of the species in 19 countries of Central and South America by the 1960s (Schliessman and Calheiro, 1974). However, a suspension or reduction in control efforts after 1965, due to the costs of the programme activities and questions concerning the necessity or feasibility of eradication, was followed by the re-infestation by *Ae. aegypti* in most of these territories (Schliessman and Calheiro, 1974). In the USA, a Public Health Service programme to eradicate *Ae. aegypti* was initiated by the Communicable Disease Center (CDC) with funds appropriated by Congress in October 1963 (Morlan and Tinker, 1965). Subsequently the range of *Ae. aegypti* has retracted but elimination has not been achieved. The vector species is now extending its range again, in particular in south-central and south-western states (Hahn et al., 2017), not only in areas where the species was historically absent in the 1960's but also in Florida (Parker et al., 2019).

In south-east Asia, where the introduction of *Ae. aegypti* is considered more recent (late 19th century; Powell and Tabachnick, 2013), World War II resulted in an enormous increase of *Ae. aegypti* populations due to the destruction of cities, the need to house refugees, and the disruption of local public health and sanitation systems (Halstead, 2006). More recently, strong economic growth coupled with improved housing standards and vector control programmes have reduced *Ae. aegypti* populations in many countries (Halstead, 2006).

Finally in Australia, human behavioural changes in water storage practices (particularly a move from rainwater tanks to piped water supplies) has probably contributed to the regression of *Ae. aegypti* north into the warmer and more tropical regions (Jansen and Beebe, 2010).

Assessing and managing the risk for vector-borne diseases (VBDs) requires solid data about the presence and absence of the respective vectors or the likelihood of introduction, establishment, spread, and proliferation (Braks et al., 2011; Sedda et al., 2014). Since these data sets are incomplete at continental scale, much effort has been invested in building spatial models of current or future distributions, spread, and even abundance of many major vector species (Caminade et al., 2012; European Centre for Disease Prevention and Control, 2009; Kraemer et al., 2019; Liu-Helmersson et al., 2019; Wint et al., 2020a; Wint et al., 2020b). For *Ae. aegypti* in particular, the species occurs only at the eastern margin of continental Europe and thus available estimates of potential distributions within Europe are based on presence/absence data, or environment and climatic limits, of currently colonised areas outside Europe (Kraemer et al., 2015b; Rogers et al., 2006).

In this study, we re-evaluate the likelihood of establishment of *Ae. aegypti* in Europe and neighbouring areas by considering historical presence data for the western Palaearctic region. Based on historical distribution presence data retrieved from the literature and museum collections, matched with historical climate data sets, we use widely established spatial modelling techniques to identify the historical suitability in Europe and project this to current and future distributions in Europe and the USA where the species has persisted since the 18th century and continued to spread while it has disappeared from the Mediterranean Basin. It should be noted that this paper is not intended to investigate the drivers or their specific impact, thus models are essentially used to provide statistical pattern matching and not explanations of cause and effect.

## 2 Material and methods

### 2.1 Mosquito occurrence data

The *Ae. aegypti* historical (<1980) European distribution data collection was obtained through an online literature search in Ovid MEDLINE®, CAB direct, and Web of Science, without year and language restrictions applied to publication date.

Search terms included dengue, yellow fever and their vectors in countries of Europe, the Caucasus, Near East and northern Africa [title:(aegypti OR fasciata OR calopus OR argenteus) AND title:(dengue OR “yellow fever” OR distribution OR presence OR occurrence OR report OR spread OR dispersion OR introduction OR “risk map” OR model$ OR climat$ OR global$) AND title:(Mediterrane$ OR Europ$ OR Balkan OR Scandinavia$ OR Iberian OR Aland OR Albania OR Andorra OR Austria OR Belgium OR Benelux OR Bosnia OR Herzegovina OR Bulgaria OR Croatia OR Cyprus OR Czech OR Denmark OR Germany OR Spain OR Estonia OR Finland OR Faroe OR France OR Greece OR Hungary OR Ireland OR Eire OR Italy OR Kosovo OR Latvia OR Liechtenstein OR Lithuania OR Luxembourg OR Macedonia OR Malta OR Montenegro OR Netherlands OR Norway OR Poland OR Portugal OR Slovenia OR Romania OR “San Marino” OR Serbia OR Slovakia OR Switzerland OR Sweden OR “United Kingdom” OR “British Isles” OR “Great Britain” OR Wales OR England OR Scotland OR Turkey OR Yugoslavia OR Armenia OR Belarus OR Bielorussia OR Georgia OR Moldova OR Ukrain$ OR Ukrayina OR Russia$ OR USSR OR SSSR OR “Soviet Union” OR Azerba$ OR Azarba$ OR Turkmen$ OR Uzbek$ OR Kyrgyz$ OR Tajik$ OR Tadjik$ OR Kazak$)]. This search was first performed in the frame of a study on dengue in Europe mandated by WHO (Schaffner and Mathis, 2014) and completed by additional reference tracking. Some historical disease and vector occurrence reviews and maps were identified but their accuracy was often not sufficient for data extraction and therefore we tracked references to trace the original *Ae. aegypti* occurrence reports. Mosquito presence data were extracted when *Ae. aegypti* (or its synonyms *Ae. argenteus*, *Culex calopus* and *Stegomyia fasciata*) was mentioned as collected or observed at a location. Geographical coordinates (latitude/longitude) were then retrieved based on location name via Google Earth and associated to the date of observation when specified or to the date of publication. No obvious geographical bias in reporting was observed: some areas show substantial inland records (e.g. Greece, Spain, Turkey) whilst others show mainly/only coastal records, but in these cases inland areas can be considered less suitable for the mosquito, because of elevation or climate (e.g. France, the Balkan's Adriatic coast).

Modern (≥1980) mosquito distribution data for the western Palaearctic region have been collected routinely within the VectorNet project via online systematic literature search, reference tracking, and grey literature and unpublished dataset sharing (Braks et al., 2022). For both the historical and modern periods, mosquito introductions without evidence of establishment were excluded from the dataset. Modern records which could not be substantiated following information exchanges with the authors or with local colleagues were also excluded (Algeria (Aïssaoui and Boudjelida, 2017; Dahchar et al., 2017), Lebanon (Knio et al., 2005)). Modern vector distribution data for the USA was taken from the datasets described earlier (Kraemer et al., 2015a).

### 2.2 Modelling

The spatial distribution modelling was performed using both Random Forest and Boosted Regression Trees to model presence and absence, implemented through the VECMAP® Software Suite (AVIA-GIS, Belgium), to produce estimates of the probability of presence. Ten replicates of each method, with a 25 % holdback, were run, and the results averaged to produce an ensemble mean. These methods require approximately equal numbers of presence and absence points to be offered to each modelling run. The occurrence data did not include the requisite absence points, so these needed to be generated. There are a number of geostatistical ways absences can be generated, but we chose to infer absences based on environmental suitability, by assigning absences to areas within 6° north and south of the known vector presence records that can be defined as biologically unsuitable for the vector as described elsewhere (Schaffner et al., 2016). This buffer restricted the model to areas reasonably close to the training data.

A location was defined as unsuitable at elevations higher than 1300 m.a.s.l. (the highest presence record in the literature was 1290 m.a.s.l., in Turkey (Irfan and Vogel, 1927)), with precipitation of <300 mm per year, with a minimum temperature of <14 °C for >6 months per year, with a maximum temperature of >39 °C for 2 months or more, and finally with a human population density of <5 people per square kilometre (Kraemer et al., 2015a).

It should be noted that these environmental limits, especially the rainfall, may falsely exclude urban or desert areas where open water is artificially maintained. For the definition of presence, the 1910 decade was taken as the historical reference point, being the first decade with significant numbers of records, and to ensure that the ‘historical’ baseline was as far apart from the present as the data justify. We assumed that there was no new expansion after 1910 and that the range retraction did not start before 1940. Any locations with presence before that baseline was therefore assumed to still be present in 1910, and data presences for later dates were assumed to be present in 1910. This study therefore assumes that 1910 represents the maximum range, and further focusses on binary presence and absence rather than abundance.

Several models were implemented using (a) historical distributions from the western Palaearctic region with 1910 climate data, and projected forward to 2015 and 2050, and (b) modern (2015) distributions for the USA, with 2015 climate, hind casted with the 1910 climate, and projected to 2050. All model outputs were global.

Projections and hind casts were made by running the base model with the covariates from the matching dates. VECMAP estimates the model parameters for the input sample points, stores these and then applies the models to the raster image covariates pixel by pixel. This means that a model can be prepared using the matching covariates, but then applied to covariates from a different time period. For this to be valid, covariates from the different periods must be compatible; it would not be appropriate to use e.g. remotely sensed temperature as contemporary covariates while using temperature based on weather station for future or past estimates.

Models were run with and without human population density as a covariate. As *Ae. aegypti* is highly anthropophilic, human density can be used as a proxy to the occurrence of suitable breeding sites with artificial watering. Models including human population density are therefore likely to be better predictors of the actual distribution, whilst the models omitting the human population should provide an indication of the climatic envelope limiting the vector's distributions. As the human population is largely concentrated in settlements, the models incorporating populations are likely to be patchy and often with such fine details that cannot easily be distinguished on continental-scale maps. Models without human population as a covariate may therefore be easier to read.

The output distribution models were evaluated using (a) area under the receiver operating characteristic (ROC) curve (AUC) using an online calculator (Eng, 2017), or (b) Cohen's Kappa. For both, a value of 0.8 or above indicates excellent agreement between sample and model. For Kappa, values of 0.4–0.6 indicate ‘moderate’ agreement.

### 2.3 Model covariates

The covariates used for spatial modelling were those for which we were able to obtain data for past, present and future periods, namely monthly minimum and maximum temperature, total monthly precipitation, monthly minimum relative humidity and human population. These climatic and demographic variables have successfully been used in modelling current and future distributions of *Aedes* species (Kraemer et al., 2019).

### 2.4 Climate data

Mean monthly temperature (minimum and maximum), precipitation (total) and relative humidity data at 0.0416667 degree resolution (approx. 5 km) for 2015 and 2050 were taken from the Worldclim or derived projected climate datasets (Kraemer et al., 2019).

Historic climate data for these parameters images were calculated for the years 1910 to 1960. The Climate Research Unit (CRU) at East Anglia have produced historic mean monthly world coverages at 0.5° resolution for these years (Harris et al., 2014). We decided to use the data from WorldClim 1.3 (Hijmans et al., 2005) as a template for pattern scaling the CRU datasets, and we used the data in the form of MetGrid files (Jones, 2019).

We constructed the mean 0.5° grids for rainfall, mean temperature and diurnal temperature range from the MetGrid files and subtracted them from the annual CRU data to create anomaly grids for each year. These anomalies were then interpolated to 2.5 min using a regular grid bicubic interpolation. Note that this was not a spline, as the derivatives were not constrained to equality at the edge of the kernel; however, tests on the data showed that the effect was not noticeable.

The interpolated anomalies added back to the 2.5 min data as tmax = tmean + diurnal range/2 and tmin = tmean - diurnal range/2; although historically mean temperature was calculated by many complicated formulae, the present WMO definition is (tmax+tmin)/2.

Neither MetGrid nor CRU data include relative humidity. The CRU data do include vapour pressure, but MetGrid and WorldClim do not; this means that there is no scaling template. Even more, the CRU images look to lack definition, which is not surprising, as they must have been constructed from sparse data.

The relative humidity images are therefore constructed from the dewpoint as follows:

${\mathrm{Rh}}_{\min}=\frac{v_{d}}{v_{x}}\cdot 100$ where *v*_*d*_ is the vapour pressure at the dewpoint, and *v*_*x*_ is vapour pressure at ambient temperature. The dewpoint can be estimated according to Linacre (Linacre, 1977) from the mean temperature and diurnal range. We used this to derive the relative humidity coverages from the previously constructed tmax and tmin.

### 2.5 Human population data

Historic population data was taken from the Hyde historical population datasets (Klein Goldewijk, 2017a; Klein Goldewijk et al., 2011), and the future population density from PBL IMAGE datasets (Klein Goldewijk, 2017b), based on the Shared Socio-economic pathway SSP2 median assumption scenarios (van Vuuren et al., 2017).

## 3 Results

**Fig. 1:**
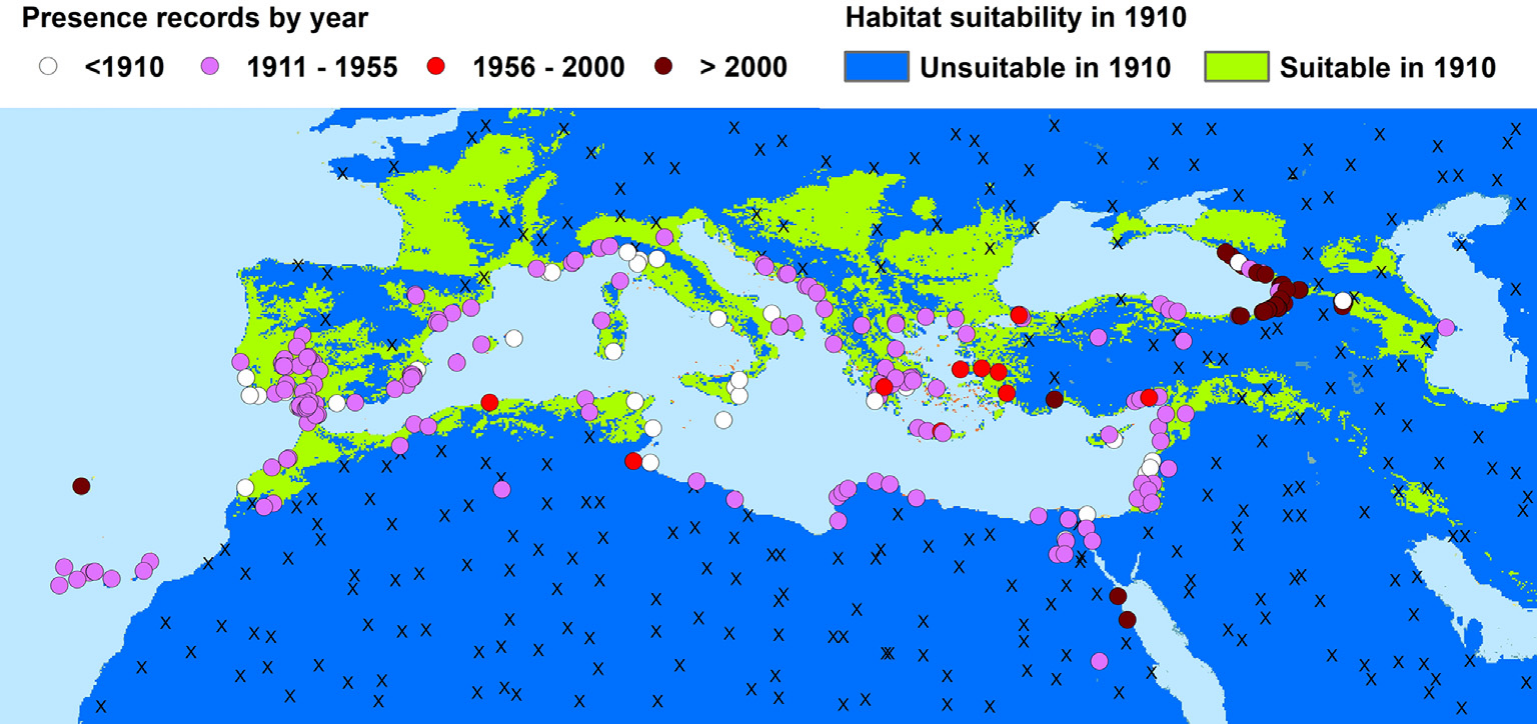
Reported occurrences of *Aedes aegypti* in the western Palaearctic region for different periods, with inferred absences and calculated suitability for the 1910 climate. Dots: presences, according to a period (colour); Crosses: Calculated absences for the historical period (up to 1955); Dots can overlap.

A total of 263 distribution data presence points of *Ae. aegypti* were gathered for the western Palaearctic region (WPR), distributed over 24 countries and comprising 213 historical and 50 modern data (Table S1, Figs. 1 and S1). They were extracted from 90 documents (containing original data), 3 museum entomological collections, 3 unpublished datasets and 1 personal communication; the detailed dataset with references can be downloaded from Figshare (Schaffner, 2022). The earliest occurrence record dates back to 1839 (Macquart: Canary Islands, as *Culex calopus* (Christophers, 1929)). The end of the ‘historical’ period is fixed at 1955, when the species was considered as disappeared in almost all countries of the WPR. Occurrences reported posterior to 1955 are considered ‘modern’ data. These included a few sporadic observations reported over the period 1955–2000, and populations established since 2001 which have been observed to breed over more than one season in previously-free areas. Our historical data set used for modelling includes all historical data we had at the time of the modelling work, by April 2021, from 1839 to 1955.

**Fig. 2:**
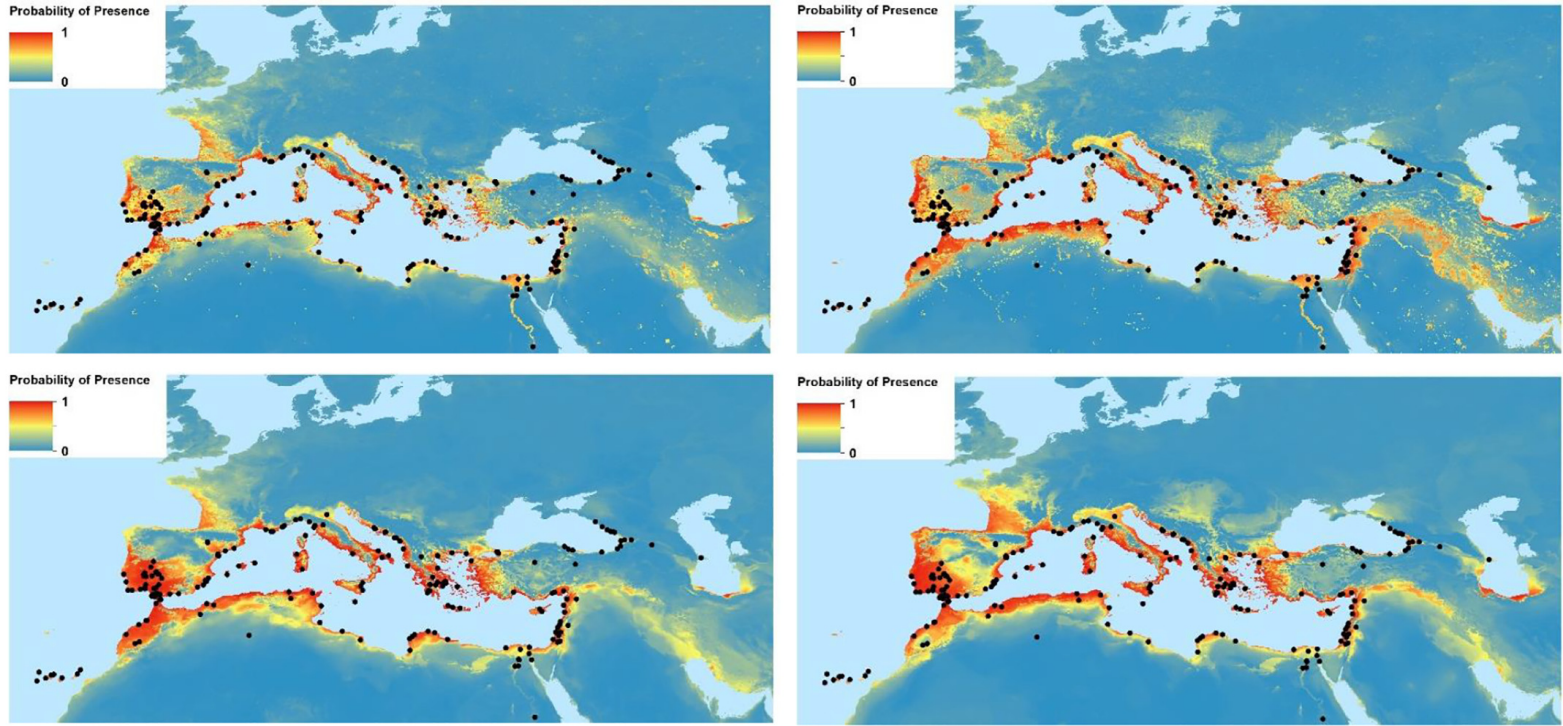
Western Palaearctic region historical 1910 (left) and 2015 (right) modelled suitability for *Aedes aegypti* with population (top) and without population (bottom) in the covariates. Black dots: recorded historical (up to 1955) presence points.

The models of both historical WPR (Fig. 2) and modern USA distributions are statistically very reliable, both with AUC >0.8 and kappa >0.5 without considering human population, and >0.7 including human population (Table 1). This indicates that the modelling process provides an accurate output for both sets of data, and that the parameters used are indeed appropriate to the task for both historical and modern time periods and both WPR and USA geographies.

**Table 1:** Accuracy metrics for the range of spatial models run. WPR: western Palaearctic region.

Vector Location Records	Cast^a^ time period	Covariate set	AUC WPR 1910 points	Kappa WPR 1910 points	AUC USA 2015 points	Kappa USA 2015 points
WPR Historical	1910	With population	0.998	0.956	0.941	0.593
WPR Historical	1910	Without population	0.994	0.924	0.815	0.435
WPR Historical	2015	With population	0.971	0.862	0.980	0.805
WPR Historical	2015	Without population	0.956	0.772	0.814	0.481
WPR Historical	2050	With population	0.964	0.800	0.981	0.873
USA 2015	1910	With population	0.917	0.714	0.980	0.813
USA 2015	1910	Without population	0.822	0.492	0.943	0.745
USA 2015	2015	With population	0.936	0.741	0.999	0.965
USA 2015	2015	Without population	0.828	0.512	0.996	0.745

a Contemporary (now) cast, hind cast or forecast using covariates from specified time period.

The historical WPR model demonstrates that historically (1910) the region was climatically suitable for the vectors well beyond the sample locations (Fig. 2, left). The suitability projected to 2015 (Fig. 2, right) suggests that the potential distribution of the vector has expanded since 1910, with the warming of the climate (Fig. 2, right bottom) and the increase in human population and warming (Fig. 2, right top). Quite large areas of southwest France, Greece and western Turkey are shown to be suitable in 2015. For 2050 this expansion pattern of suitability is projected to continue (Fig. S2, bottom), further highlighting Turkey and Greece, and the Near East, North Africa, and western France. Climatic variability and extreme events may also affect the vector presence, but they are not reflected by the simple climatic levels used here.

Accuracy metrics (Table 1) also suggest that the extrapolated historical WPR model provides a reasonable to good fit to the current 2015 USA distributions (AUC >0.8, Kappa >0.44), and is also substantially better with population in the covariate suite than without. Perhaps somewhat less expected is that the USA model's prediction for historical WPR also appears to work reasonably well. The agreement between the hindcast and spatially extrapolated contemporary USA data and the historical WPR samples points is also close (Table 1, rows 7–10, column 4, AUC >0.8, with and without population in covariates).

## 4 Discussion

These results show that the available historical climatic and demographic correlates can be used to successfully produce spatial models of historical *Ae. aegypti* distributions. Forward projections of these models suggest that areas with conditions suitable for the vector to survive and prosper have expanded, and they will continue to expand in the future at least as far as 2050. Models built using historical WPR distribution data can be reliably extrapolated to predict current vector presence in the USA, and models built using current USA vector distribution data extrapolate well to predict the historical distribution in the WPR. These results suggests that the approach of Rogers and Hay (European Centre for Disease Prevention and Control, 2012), who inferred the suitability of European habitats to *Aedes* vectors based largely on vector samples from outside Europe, was reasonable. When projected to 2050, the predicted WPR suitability for the vector (Table 1 and Fig. S2) is shown to expand further, and it does not suggest that the future environment is any less suitable for the vector than it is now. This concurs to some extent with the findings of other authors who have projected the *Aedes* distributions into the future (Kraemer et al., 2019, Trájer, 2021 #457; Liu-Helmersson et al., 2019).

We are left wondering why *Ae. aegypti* has not filled this large niche predicted suitable by the models in the WPR for the current climate, whereas it continues to spread in the parts of the USA which, as we have shown, has similar habitat suitability. Since the 1950's, the only records of present-day established populations in the WPR are in Madeira, along the eastern Black Sea coast and in southern Egypt, while introductions occurred at Points of Entry. With regards to the huge surveillance effort implemented while targeting the invasive species *Ae. (Stegomyia) albopictus* (Skuse, 1894), the distribution of *Ae. aegypti* cannot be considered underestimated in the WPR (Wint et al., 2022, In prep.).

There are a number of potential explanations why the vector has failed to re-establish in continental western and southern Europe as discussed below:

a) The models based on European historical distributions overestimate suitability. Yet the suitability inferred by models trained with very distinct North-American and European distributions are similar which suggests that the models are realistic.
b) Conditions at the reported Points of Entry are unsuitable or introductions occur at the wrong time of the year. Whilst this might be the case, there likely were plenty of potential introductions in other suitable areas that have not specifically been monitored, and still did not yet result in the establishment of populations in the surrounding areas. Models suggest ports of southern Europe (e.g. Algeciras and Barcelona, Spain) to be suitable for *Ae. aegypti* establishment, high local densities and initial dispersal (Da Re et al., 2021; Trájer, 2021).
c) The more-or-less universal presence of piped water in Europe reduces the availability of larval breeding sites. However, the USA has a similar omni-presence of piped water, and this does not prevent the mosquito from thriving. Furthermore, relationships between socioeconomic factors and the distribution and abundance of *Ae. aegypti* in mainland USA is found to be inconsistent (Holeva-Eklund et al., 2021). This also weakens the hypothesis that the development of piped water distributions was a major contributor to the disappearance of *Ae. aegypti* from Europe.
d) There is sufficient mosquito control (this includes control of urban mosquito species by public agencies, by pest control companies or by citizen indoor spraying) at Points of Entry and in urbanised areas in Europe to prevent the establishment of *Ae. aegypti* after introduction. However, the establishment of other container-breeding invasive species (e.g. *Ae. albopictus*) throughout Europe could not be prevented. Most mosquito control efforts in Europe focus on floodplains rather than habitats suitable for *Ae. aegypti*. Furthermore, mosquito abatement programmes in the USA are arguably more extensive than in Europe and have been in place since the early 1900's, yet the vector continues to persist.
e) There is interspecific competition between *Ae. aegypti* and other mosquito species, particularly *Ae. albopictus* and perhap*s Culex (Culex) pipiens* Linnaeus, 1758. Though there are suggestions that the two *Aedes* container-breeding species do compete in terms of larval habitat colonisation and that asymmetric satyrisation of *Ae. aegypti* females by *Ae. albopictus* males may prevent establishment or result in a displacement of *Ae. aegypti*, it is also suggested that locally variable climate-driven mortality of *Ae. albopictus* eggs and rapid evolution for resistance to cross-mating may mitigate a competitive superiority (Burford Reiskind et al., 2018; Lounibos et al., 2010). As observed, *Ae. albopictus* would not dominate under all conditions and thus not fully displace *Ae. aegypti*. Further, these two species do coexist in the USA and at other places such as Brazil (Braks et al., 2003; Lounibos et al., 2016) and the range of *Ae. aegypti* continues to change, with ongoing re-establishment in southern USA and even expansion in other counties (Hahn et al., 2017; Monaghan et al., 2019). Finally, given that none of the other known invasive species, in particular *Ae. albopictus*, appeared before the mid-1970's and none occupied the predicted areas of suitability until decades later, we cannot expect any impact of other invasive species on the re-establishment of *Ae. aegypti* until very recently. This leaves plenty of time for some re-establishment to have occurred in the Mediterranean Europe before *Ae. albopictus* spread there.
f) *Aedes aegypti* with a potential to invade and establish in Europe is phenotypically different from those established elsewhere. This seems unlikely as the invasive form of this vector was shown to be a single lineage (Brown et al., 2011; Powell and Tabachnick, 2013) which has spread globally, continues to invade in the USA where the habitat suitability is comparable to Europe. Also, there are substantial trade and travel movements from the USA to Europe, and the mosquito population which was historically widespread in Europe disappeared.
g) There are not sufficient suitable breeding sites in Europe. This can be discounted as the environmental habitats are shown to be suitable, and other container breeding species are doing well in Europe.
h) Even if successfully introduced, *Ae. aegypti* cannot spread. Given the rapid spread of *Ae. albopictus* in southern Europe, this seems unlikely. However, at more northern locations, e.g. Germany, *Ae. aegypti* is certainly unable to establish while *Ae. albopictus* is present thanks to its diapausing eggs that are adapted to more temperate climate (Lounibos et al., 2003). At such northern locations, e.g. Saint-Nazaire, France, or Swansea, UK, historical vector presence and yellow fever outbreaks were solely reported aboard ships or within a port city. Both vector populations and pathogen transmission did not persist to the next year (Aeger, 1902). This suggests that failure to overwinter may have prevented spread. The climate has, however, become more suitable for the mosquito since then, and although the winter temperature constraint still applies to the northern latitudes it is less but less of a constraint in the warmer Mediterranean region.
i) During the modern period, *Ae. aegypti* has not been imported into Europe often enough or in sufficient numbers to establish. Propagule pressure has been considered to have favoured the mosquito's invasion success (Lounibos, 2002). However, this seems unlikely as it has not applied to the other invasive species and there is considerable trade and travel to Europe from countries that harbour *Ae. aegypti* (including the USA). Further, the vector has been recorded at EU Points of Entry, and has established itself in Madeira after a single founder introduction event via a modern ship or aircraft (Seixas et al., 2019). Models suggest that a small quantity of eggs (10−1000) have the potential to cause establishment (Da Re et al., 2021).
j) Though there are substantial cargo and travel movements, they are of the wrong type to carry enough *Ae. aegypti* to establish new populations at points of introduction. A decrease of mosquito presence on ships was reported with the change from wood to iron hulls, which led to the reduction, if not disappearance of water in the ship hold (in particular the bilge water) (Chantemesse and Borel, 1905; Theobald, 1911). Several publications, however, point to the fact that modern marine cargo shipments are relatively efficient at spreading *Ae. aegypti* (Fonzi et al., 2015) and they obviously survive intercontinental flights (Ibáñez-Justicia et al., 2020). Indeed, recent genomic data from Indo-Pacific suggest the incursion pathways of *Ae. aegypti* into Australia were mostly linked to aerial routes from tourism hotspots (Schmidt et al., 2020).
k) A combination of any or all of the above prevented the vector re-establishing in continental western and southern Europe.

## 5 Conclusions

In summary, most of the putative reasons why *Ae. aegypti* has not re-emerged in Europe appear to be contradicted by the evidence of it thriving in the USA in broadly similar climatic and economic conditions. The vector distributions in the US are likely to have been affected by control efforts and land use changes, and to some degree by interaction with other vectors (e.g. *Ae. albopictus*), but this has not resulted in the widespread and complete disappearance that occurred in Europe. Was it really development of piped water supply systems and vector control that eliminated *Ae. aegypti* in Europe in the 1950's? What has prevented the *Ae. aegypti* adapted to USA conditions from establishing in Europe? Perhaps the most likely single reason is that the vector requires significant numbers to establish, and these are only feasible from ships carrying large numbers of people, with sufficiently humid conditions, i.e. with standing water bodies allowing reproduction during the weeks of travel, and with enough containers to sustain large populations. Relatively few of these exist today in the era of air-conditioned cabins, watertight freight containers, and improved sanitation, and thus the number of opportunities for introductions may, as yet, have been insufficient for a population to establish.

In the authors' opinion, it remains largely unanswered why *Ae. aegypti* has not re-colonised southern Europe despite suitable conditions as revealed by our models and it seems likely it is a combination of all the factors mentioned in our discussion rather that any single reason. It behoves the scientific community to delve further into this question as it may uncover limiting factors that can be used to mitigate the spread of the yellow fever mosquito in a future with increasing global trade and expanding global presence of the vector providing more chances of establishment, and with warmer winters and more intense precipitation providing more suitable larval habitats.

## CRediT authorship contribution statement

**Neil Alexander:** Validation, Formal analysis, Investigation, Visualisation. **Peter Jones:** Validation, Data curation, Writing – review & editing. **Moritz Kraemer:** Validation, Data curation, Writing – review & editing. **Francis Schaffner:** Conceptualisation, Methodology, Data acquisition and curation, Writing – original draft, Visualisation. **William Wint:** Conceptualisation, Methodology, Validation, Formal analysis, Investigation, Data curation, Writing – original draft, Visualisation.

## Declaration of competing interest

The authors declare that they have no known competing financial interests or personal relationships that could have appeared to influence the work reported in this paper.

## Appendix A Supplementary data

Supplementary material: Table S1, Figs. S1–S2 Image 1
