# Supplementary material for: Past, present and future distribution of the yellow fever mosquito Aedes aegypti: The European paradox
Source: Sci Total Environ. 2022 Nov 15;847:157566. doi: 10.1016/j.scitotenv.2022.157566 (PMC9514036; doi:10.1016/j.scitotenv.2022.157566)

Supplementary material to:

**Past, present and future distribution of the yellow fever mosquito *Aedes aegypti*: The European paradox**

William Wint, Peter Jones, Moritz Kraemer, Neil Alexander & Francis Schaffner

Table S1. Numbers of presence point data for *Aedes aegypti* per country of the western Palaearctic region and per time period, gathered from the literature, entomological collections, unpublished data sets and personal communications. Mosquito introductions without evidence of establishment and not substantiated recent records are not included.

|  | **Historical data** | | | | | | | **Modern data** | | | | |
| --- | --- | --- | --- | --- | --- | --- | --- | --- | --- | --- | --- | --- |
| Country | 1839-1900 | 1901-1910 | 1911-1920 | 1921-1930 | 1931-1940 | 1941-1955 | **Total H. d.** | 1956-1960 | 1961-2000 | 2001-2020 | **Total C. d.** | **Grand Total** |
| Albania |  |  | 1 |  |  |  | **1** |  |  |  | **0** | **1** |
| Algeria | 1 | 2 | 1 | 1 |  | 2 | **7** |  |  |  | **0** | **7** |
| Azerbaijan |  |  |  | 1 |  |  | **1** |  |  |  | **0** | **1** |
| Croatia |  |  |  | 4 |  |  | **4** |  |  |  | **0** | **4** |
| Cyprus |  | 1 |  |  |  | 1 | **2** |  |  |  | **0** | **2** |
| Egypt |  | 3 | 3 | 4 |  |  | **10** |  |  | 2 | **2** | **12** |
| France | 1 | 2 | 3 | 1 |  |  | **7** |  |  |  | **0** | **7** |
| Georgia |  |  | 1 | 6 | 2 |  | **9** |  |  | 17 | **17** | **26** |
| Gibraltar | 1 |  |  |  |  |  | **1** |  |  |  | **0** | **1** |
| Greece |  | 2 | 6 | 9 | 3 | 5 | **25** | 1 |  |  | **1** | **26** |
| Israel |  |  | 4 |  |  |  | **4** |  |  |  | **0** | **4** |
| Italy | 10 |  | 3 | 2 | 2 |  | **17** |  | 1 |  | **1** | **18** |
| Lebanon |  | 3 |  |  |  |  | **3** |  |  |  | **0** | **3** |
| Libya |  |  |  | 8 |  | 1 | **9** |  |  |  | **0** | **9** |
| Malta |  | 1 |  |  |  |  | **1** |  |  |  | **0** | **1** |
| Montenegro |  |  |  | 2 | 1 |  | **3** |  |  |  | **0** | **3** |
| Morocco |  | 1 | 3 | 1 | 2 |  | **7** |  |  |  | **0** | **7** |
| Palestine |  |  | 1 | 1 |  |  | **2** |  |  |  | **0** | **2** |
| Portugal-Mainland |  | 3 |  | 2 | 1 |  | **6** |  |  |  | **0** | **6** |
| Portugal-Madeira |  |  |  |  |  |  | **0** |  |  | 1 | **1** | **1** |
| Russia |  |  |  | 1 | 1 |  | **2** |  |  | 5 | **5** | **7** |
| Spain (incl. Canaries) | 1 | 6 | 3 | 47 | 8 |  | **65** |  |  |  | **0** | **65** |
| Syria |  |  | 3 | 1 |  |  | **4** |  |  |  | **0** | **4** |
| Tunisia |  | 3 |  |  | 1 |  | **4** |  |  |  | **0** | **4** |
| Turkey | 1 |  | 3 | 14 |  | 1 | **19** |  | 5 | 18 | **23** | **42** |
| **Total** | **15** | **27** | **35** | **105** | **21** | **10** | **213** | **1** | **6** | **43** | **50** | **263** |

The dramatic increase of data (modern *versus* historical data) in Georgia, Russia and Turkey is due to the implementation of specific mosquito surveillance following the first report of *Ae. aegypti* presence in the region (Yunicheva et al., 2008), partly as results of VectorNet field missions (Akiner et al., 2006).”

Figure S1. Allotment of gathered historical (by 1955) and modern (posterior to 1955) presence point data for *Aedes aegypti* per country of the western Palaearctic region.

Figure S2. Modelled historical (1910; top) and projected future (2050; bottom) suitability for *Aedes aegypti* (with population included in covariates) for the western Palaearctic region. Black dots: historical presence records.


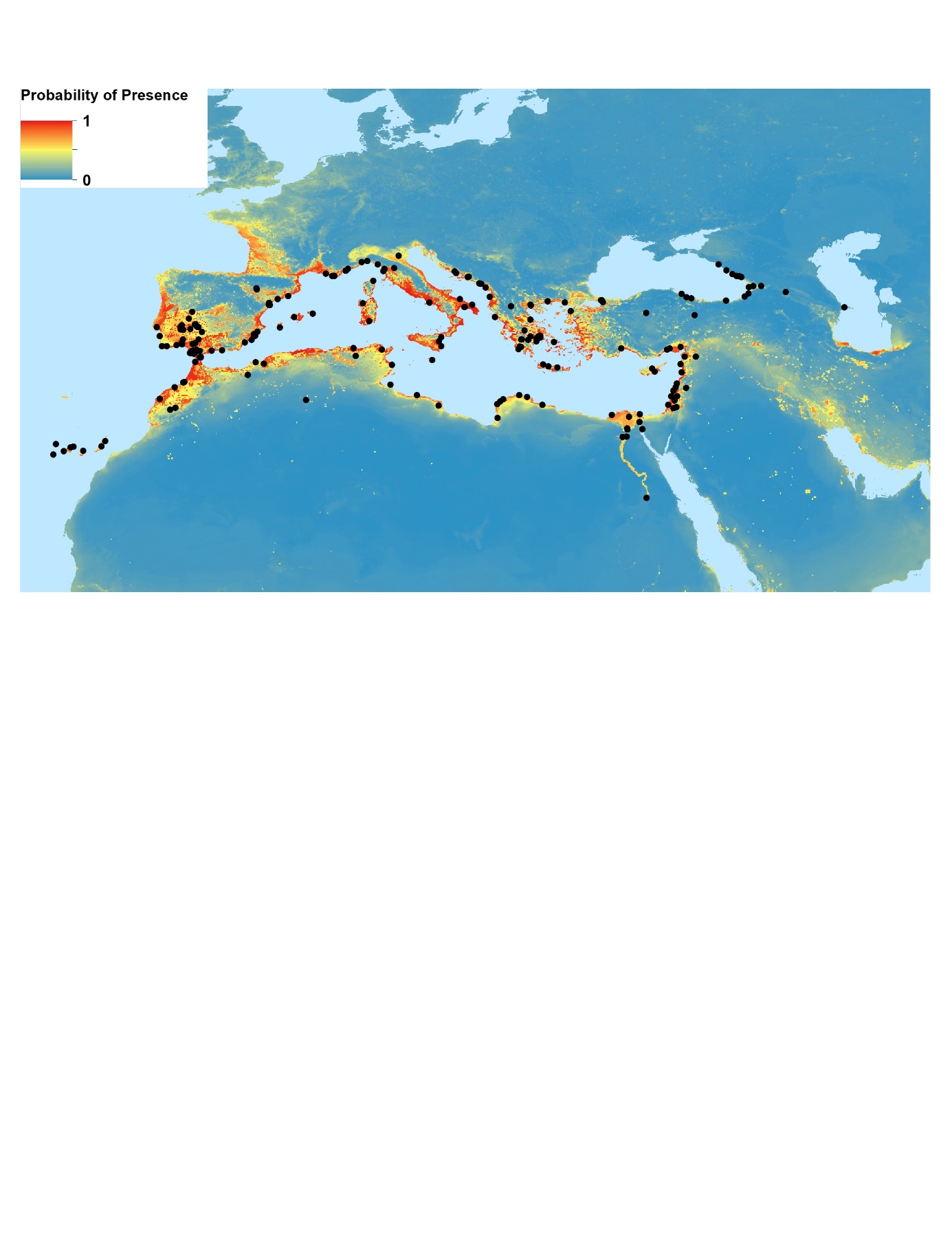

Supplement: Supplementary file 1 — Supplementary material: Table S1, Figs. S1–S2 [file mmc1.docx]
